# Cumulative incidence and risk factors of dementia in Parkinson's disease and parkinsonism: a population-based study

**DOI:** 10.1093/braincomms/fcag330

**Published:** 2026-08-28

**Authors:** Lorenzo Malfer, Aidan F Mullan, Elena Lynott, Colton Troxel, Farwa Ali, Eduardo E Benarroch, Bradley F Boeve, James H Bower, Rodolfo Savica

**Affiliations:** Department of Neurology, Mayo Clinic, Rochester, MN 55905, USA; Department of Health Sciences Research, Mayo Clinic, Rochester, MN 55905, USA; Department of Neurology, Mayo Clinic, Rochester, MN 55905, USA; Department of Neurology, Mayo Clinic, Rochester, MN 55905, USA; Department of Neurology, Mayo Clinic, Rochester, MN 55905, USA; Department of Neurology, Mayo Clinic, Rochester, MN 55905, USA; Department of Neurology, Mayo Clinic, Rochester, MN 55905, USA; Department of Neurology, Mayo Clinic, Rochester, MN 55905, USA; Department of Neurology, Mayo Clinic, Rochester, MN 55905, USA; Department of Health Sciences Research, Mayo Clinic, Rochester, MN 55905, USA

**Keywords:** parkinsonism, Parkinson’s disease, dementia, non-motor symptoms, eye movement abnormalities

## Abstract

Cognitive impairment is among the most common non-motor symptoms in parkinsonism and Parkinson’s disease, ranging from subjective complaints to dementia. Although several predictive factors have been described, their prognostic relevance remains uncertain. This study aimed to examine the predictive association between clinical features and the risk of dementia and determine the incidence of dementia in a population-based cohort of incident Parkinsonism cases. Patients with parkinsonism onset between 1991 and 2020 were identified using the Rochester Epidemiology Project records-linkage system. Subjects with cognitive impairment preceding motor symptoms onset or concomitant Alzheimer’s disease were excluded. Diagnoses of dementia and mild cognitive impairment were established using neuropsychological testing or brief cognitive screening. Development of dementia was assessed using a Cox proportional hazards regression model. Associations between dementia and each risk factor were individually estimated in adjusted Cox models. All risk factors were also included in a least absolute shrinkage and selection operator penalized multivariable Cox model. A total of 1104 patients were included: 153 (13.9%) developed mild cognitive impairment, and 543 (49.2%) developed dementia during follow-up. The cumulative incidence of dementia was 30.3% at 5 years, 48.6% at 10 years, 61.4% at 15 years and 69.3% at 20 years, indicating that a considerable portion of patients remained free of dementia after a prolonged follow-up. The majority of patients with dementia were males (61.5%), and older age at disease onset was associated with risk of dementia (hazard ratio [HR] = 1.32, *P* < 0.001 per 5-year increment). In the penalized model, dementia risk was significantly higher among individuals presenting with impaired postural reflexes (HR = 1.26, *P* = 0.014), consistent with a poorer prognosis in akinetic-rigid-predominant parkinsonism. Eye movement abnormalities, including reduced ocular range of motion (HR = 1.86, *P* < 0.001) and smooth pursuit alterations (HR = 1.34, *P* = 0.024), were also associated with dementia. Non-motor symptoms, including rapid eye movement sleep behaviour disorder (HR = 1.51, *P* < 0.001), orthostatic hypotension (HR = 1.30, *P* = 0.009) and constipation (HR = 1.24, *P* = 0.022), were linked to dementia risk, supporting a potential involvement of non-dopaminergic and central autonomic pathways in cognitive decline. These findings were largely confirmed in the Parkinson’s disease/Parkinson’s disease with dementia group, in which 77 (13.5%) developed mild cognitive impairment and 249 (43.5%) developed dementia. Among these patients, smooth pursuit alterations, reduced ocular range of motion, orthostatic hypotension and constipation were associated with dementia.

## Teaser

Malfer *et al*. report a 69.3% cumulative incidence of dementia at 20 years in a population-based parkinsonism cohort, with 67.8% in Parkinson’s disease. Impaired postural reflexes, orthostatic hypotension, smooth pursuit alterations, and reduced ocular range of motion were among the strongest associated risk factors in the entire cohort and Parkinson’s disease.

## Graphical Abstract

**Graphical Abstract:**
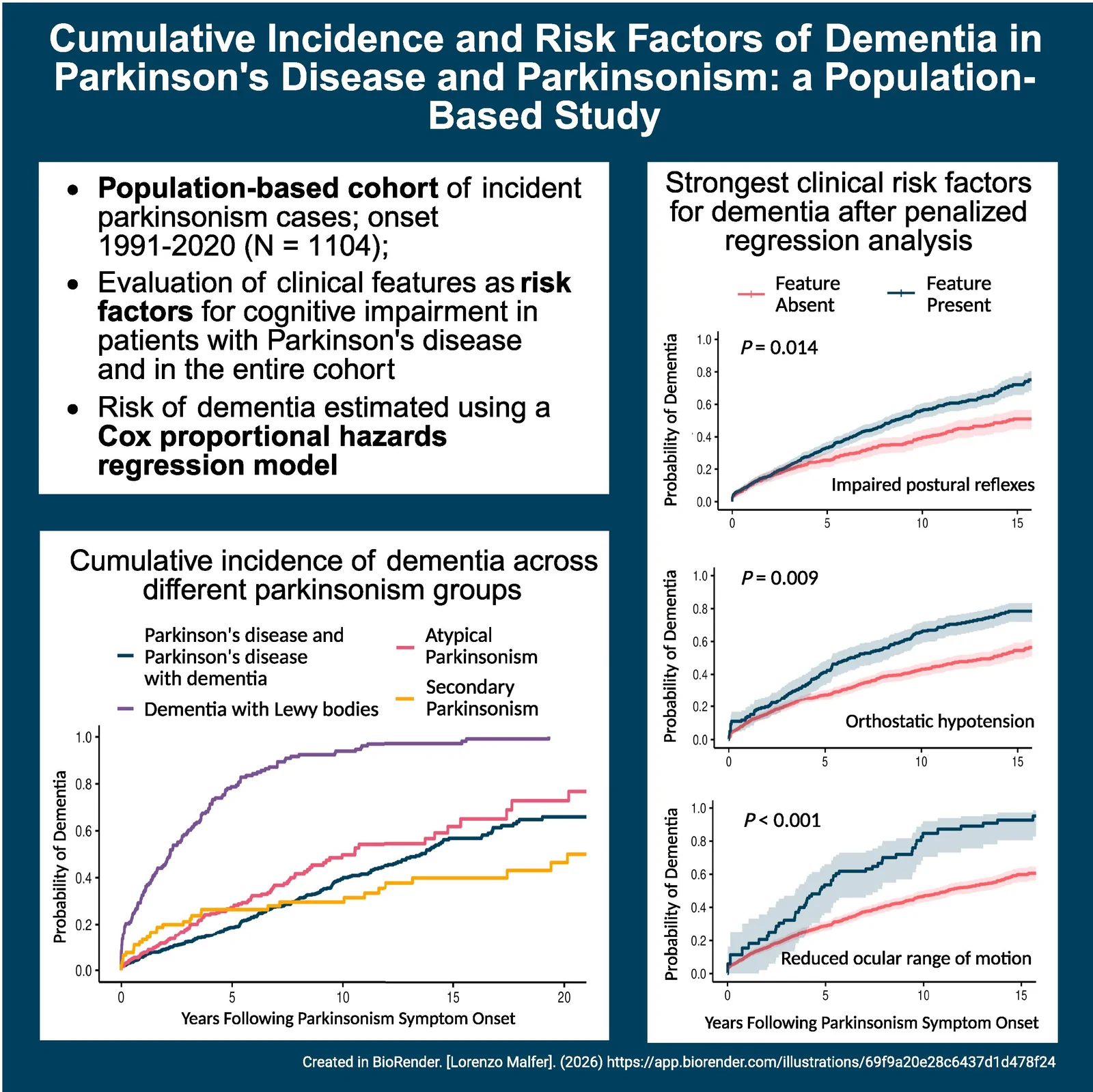

## Introduction

Parkinson’s disease (PD) is a common neurodegenerative disorder characterized by both motor symptoms, such as bradykinesia, tremor, rigidity and postural instability, and non-motor symptoms, including autonomic dysfunction, neuropsychiatric changes and cognitive impairment.^1-3^ Among non-motor manifestations, cognitive impairment is particularly prevalent, manifesting across a spectrum of severity ranging from cognitive complaints in the prodromal stages to mild cognitive impairment (MCI) or dementia.^4,5^ Dementia is particularly debilitating, as it has been linked to severe decline in quality of life, increased caregiver burden, institutionalization and mortality.^6-8^ Population-based studies on PD cohorts have reported a prevalence of MCI ranging between 20% and 60%, with 15%–20% of individuals exhibiting cognitive symptoms at the time of diagnosis.^9-12^ Although progression to dementia is not inevitable, recent prospective analyses have shown that approximately 50% of patients with PD develop dementia within 15 years of disease onset and approximately 75% within 20 years.^13^ A recent meta-analysis also reported a 4.5% annual risk of dementia in a PD population, which is 3.2 times greater than that observed in age-matched healthy controls.^14^ Moreover, cognitive impairment is a core diagnostic criterion for dementia with Lewy bodies (DLB) and is frequently observed in other atypical parkinsonism subtypes.^15^ However, estimates of long-term dementia risk vary across cohorts depending on study design, clinical setting and follow-up duration.

Clinical features of cognitive decline in parkinsonian syndromes encompass various cognitive domains, including executive function, visuospatial ability, language and memory.^16,17^ Notably, the presence of cognitive impairment in PD correlates with a widespread cortical deposition of Lewy bodies as well as the concomitant presence of Alzheimer’s pathology.^18^ Although the underlying mechanisms are not fully understood, different pathological processes have been proposed to increase vulnerability and cognitive deterioration.^17^ Additionally, several risk factors have been suggested as predictors of cognitive impairment in parkinsonism. These features include family history of dementia, lower levels of premorbid education, older age at disease onset, longer disease duration, more severe motor features, predominant postural instability or akinetic-rigid PD subtype, REM (rapid eye movement) sleep behaviour disorder (RBD), psychosis, depression and anxiety.^19-21^ Predicting which patients are at greater risk of developing dementia has several important implications and could guide prognostic risk stratification and individualized clinical and therapeutic approaches.

The primary objective of this study was to examine the predictive association between demographic and clinical features and the risk of cognitive impairment in a population-based cohort of incident parkinsonism cases. In addition, we aimed to assess the cumulative incidence of MCI and dementia across different parkinsonism subtypes.

## Materials and methods

### Study design and case ascertainment

All patients with a diagnosis of parkinsonism who were residents of Olmsted County, MN, from 1991 to 2020 were identified through the Rochester Epidemiology Project (REP) record-linkage system. A detailed description of the REP system and the methodology used for case identification and parkinsonism classification in this population has been previously reported.^22,23^

Initially, parkinsonism was defined as the presence of at least two of the four cardinal signs: rest tremor, bradykinesia, rigidity and impaired postural reflexes. Cases were subsequently classified into different diagnostic categories using established criteria. PD was diagnosed according to the diagnostic criteria by Postuma *et al*.^2^ PD dementia was diagnosed according to the criteria by Poewe *et al*.^24^ DLB was diagnosed according to the criteria by McKeith *et al*.^25^ Progressive supranuclear palsy (PSP) was diagnosed according to the criteria by Hoglinger *et al*.^26^ Multiple system atrophy (MSA) was defined by the presence of either prominent/early (<1 year) cerebellar syndrome, or prominent/early autonomic syndrome.^27^ Criteria for drug-induced parkinsonism (DIP) included all three of the following: (i) onset within 6 months of treatment with neuroleptic or dopaminergic-depleting drug; (ii) no symptoms before treatment; (iii) resolution of symptoms upon withdrawal of treatment.^28^ Criteria for surgical-induced parkinsonism (SP) included all three of the following: (i) onset within 6 months of neurosurgery, (ii) no symptoms before surgery and (iii) imaging abnormalities within relevant basal ganglia regions on computed tomography/magnetic resonance imaging (CT/MRI). Criteria for vascular parkinsonism (VP) included both of the following: (i) non-progressive or stepwise disease and (ii) evidence of an infarct in relevant basal ganglia regions on CT/MRI.^27^ Unspecified parkinsonism (UP) was defined as parkinsonism with at least one absolute exclusion criterion or red flag for PD according to the MDS clinical diagnostic criteria, without meeting criteria for any other defined parkinsonian syndrome. Parkinsonism subtypes were then classified into the following four cohorts: PD/PD with dementia (PD/PDD), DLB, atypical parkinsonism (MSA, PSP and UP) and secondary parkinsonism (DIP, VP and SP).^29^ Data were collected retrospectively, and the initial clinical diagnosis was assigned after a 5-year follow-up from symptom onset to allow adequate characterization. Diagnoses were reviewed by a movement disorder specialist and a physician; disagreements were resolved by consensus, and when consensus could not be reached, the final diagnosis was assigned by the movement disorder specialist. Formal inter-rater reliability was not systematically calculated. Diagnostic categories were not subsequently reclassified, except for PD which was reclassified as PDD when patients developed dementia over 1 year after the onset of motor symptoms.

All clinical symptoms relevant to the differential diagnosis of parkinsonism were analyzed, including cerebellar signs, postural instability, axial rigidity, wide-based gait, en bloc movements, focal dystonia, dysarthria/dysphagia, saccade abnormalities, smooth pursuit alterations, reduced ocular range of motion, RBD, orthostatic hypotension, neurogenic bladder, constipation and erectile dysfunction. In addition, imaging was reviewed for evidence of hydrocephalus and basal ganglia infarction.

Dementia and MCI diagnoses were established based on clinical evaluation by a neurologist or a different physician, supported by either formal neuropsychological testing or brief cognitive screening using Mini-Mental State Examination, Short-Test of Mental Status or Montreal Cognitive Assessment. Diagnoses were then retrospectively confirmed by a physician (L.M.). Patients with any cognitive impairment preceding the onset of motor symptoms were excluded. Based on the cognitive status, the cohort was divided into three groups: patients without MCI, patients with MCI and patients with dementia.

### Statistical analysis

No sampling procedures were involved as all eligible patients were included. Numerical characteristics were summarized with medians and interquartile ranges; categorical characteristics were summarized with frequency counts and percentages. The primary outcome of interest was the development of dementia following the onset of parkinsonian motor symptoms, which was assessed using a Cox proportional hazards regression model. Potential risk factors for dementia included motor and non-motor symptoms of parkinsonism, and brain imaging abnormalities. Patient follow-up time was censored at the date of death or at the last available follow-up. All risk factors were treated as time-dependent covariates to account for the variable development of symptoms relative to the onset of parkinsonism. As such, the temporal span between individual predictors and dementia onset varied across the cohort.

The risk of dementia associated with each risk factor was modelled individually, adjusted for age at onset of parkinsonism, sex and parkinsonism subtype (PD/PDD, DLB, atypical parkinsonism and secondary parkinsonism). Model results were reported with hazard ratios (HRs) and 95% confidence intervals (CIs). All risk factors were also included together in a least absolute shrinkage and selection operator (LASSO) penalized multivariable Cox model to identify independent predictors of dementia. The LASSO penalty was determined using 10-fold cross-validation optimized on model concordance, and all variables selected in the final model were reported. An analysis of predictors of dementia using the LASSO-penalized multivariable Cox regression model was performed in the whole cohort and in patients with PD/PDD only. Variables selected by the LASSO model were reported regardless of the *P*-values, as inclusion was determined by the penalized selection procedure rather than by significance thresholds.

All tests were two-sided, and *P*-values less than 0.05 were considered significant. Analyses were conducted using R version 4.4.1.

### Ethical approval

The Institutional Review Boards of the Mayo Clinic and the Olmsted Medical Center approved this study. Written informed consent for the use of medical information was obtained from all participants according to the Declaration of Helsinki. Individuals who denied authorization to use their medical records for research were excluded.

## Results

### Demographics

This population-based cohort included 1104 patients diagnosed with parkinsonism with motor symptom onset between 1991 and 2020. Among these, 503 patients were diagnosed with PD, 69 with PDD, 157 with DLB, 25 with MSA, 44 with PSP, 69 with DIP, 22 with VP, 2 with SP and 213 with UP. The cohort included 665 (60.2%) males, 430 (38.9%) females and 9 (0.8%) patients who preferred not to disclose this information. The median age of onset was 75 years (interquartile range [IQR]: 66–81). A similar median age of onset was observed in all subgroups, except for secondary parkinsonism, which presented a median age of 65 years (IQR: 54–77).

A complete summary of demographic characteristics, parkinsonism subtype, cardinal motor symptoms and cognitive status is provided in Table 1.

**Table 1:** Summary of patient demographics and parkinsonism disease characteristics

	All patients	PD/PDD	Dementia with Lewy bodies	Atypical parkinsonism	Secondary parkinsonism
	*n* = 1104	*n* = 572	*n* = 157	*n* = 282	*n* = 93
Age at onset, years					
Median (Q1, Q3)	75 (66, 81)	73 (66, 81)	76 (71, 81)	77 (69, 83)	65 (54, 77)
Motor symptom onset to dementia, years					
Median (Q1, Q3)	3.8 (1.2, 7.8)	5.9 (2.7, 9.7)	2.1 (0.5, 4.1)	3.4 (1.5, 6.8)	1.7 (0.6, 10.3)
Time to last follow-up or death, years					
Median (Q1, Q3)	7.5 (4.5, 11.7)	9.3 (5.6, 13.2)	5.9 (4.0, 8.9)	5.7 (3.5, 9.2)	8.4 (3.7, 16.8)
Sex, *n* (%)					
Female	430 (38.9)	217 (37.9)	57 (36.3)	103 (36.5)	53 (57.0)
Male	665 (60.2)	349 (61.0)	99 (63.1)	177 (62.8)	40 (43.0)
Not disclosed	9 (0.8)	6 (1.0)	1 (0.6)	2 (0.7)	0 (0.0)
Parkinsonism subtype, *n* (%)					
PD	503 (45.6)	503 (87.9)	—	—	—
PDD	69 (6.3)	69 (12.1)	—	—	—
Dementia with Lewy bodies	157 (14.2)	—	157 (100.0)	—	—
Progressive supranuclear palsy	44 (4.0)	—	—	44 (15.6)	—
Multiple system atrophy	25 (2.3)	—	—	25 (8.9)	—
Drug-induced parkinsonism	69 (6.3)	—	—	—	69 (74.2)
Vascular parkinsonism	22 (2.0)	—	—	—	22 (23.7)
Surgical parkinsonism	2 (0.2)	—	—	—	2 (2.2)
Unspecified parkinsonism	213 (19.3)	—	—	213 (75.5)	—
Cardinal motor symptoms, *n* (%)					
Rest tremor	757 (68.6)	455 (79.5)	98 (62.4)	143 (50.7)	61 (65.6)
Bradykinesia	971 (88.0)	528 (92.3)	130 (82.8)	230 (81.6)	83 (89.2)
Rigidity	893 (80.9)	488 (85.3)	120 (76.4)	210 (74.5)	75 (80.6)
Impaired postural reflexes	666 (60.3)	314 (54.9)	117 (74.5)	191 (67.7)	44 (47.3)
Post-motor symptom onset cognitive impairment, *n* (%)					
Mild cognitive impairment	153 (13.9)	77 (13.5)	7 (4.5)	54 (19.1)	15 (16.1)
Dementia	543 (49.2)	249 (43.5)	150 (95.5)	109 (38.7)	35 (37.6)

Source of cognitive impairment diagnosis, *n* (%)	*n* = 696	*n* = 326	*n* = 157	*n* = 163	*n* = 50
Mayo neurologist	425 (61.1)	210 (64.4)	113 (72.0)	81 (49.7)	21 (42.0)
Mayo physician	224 (32.2)	96 (29.4)	36 (22.9)	68 (41.7)	24 (48.0)
Non-Mayo physician	47 (6.8)	20 (6.1)	8 (5.1)	14 (8.6)	5 (10.0)

PD, Parkinson’s disease; PDD, Parkinson’s disease with dementia.

### Cognitive impairment

**Figure 1:**
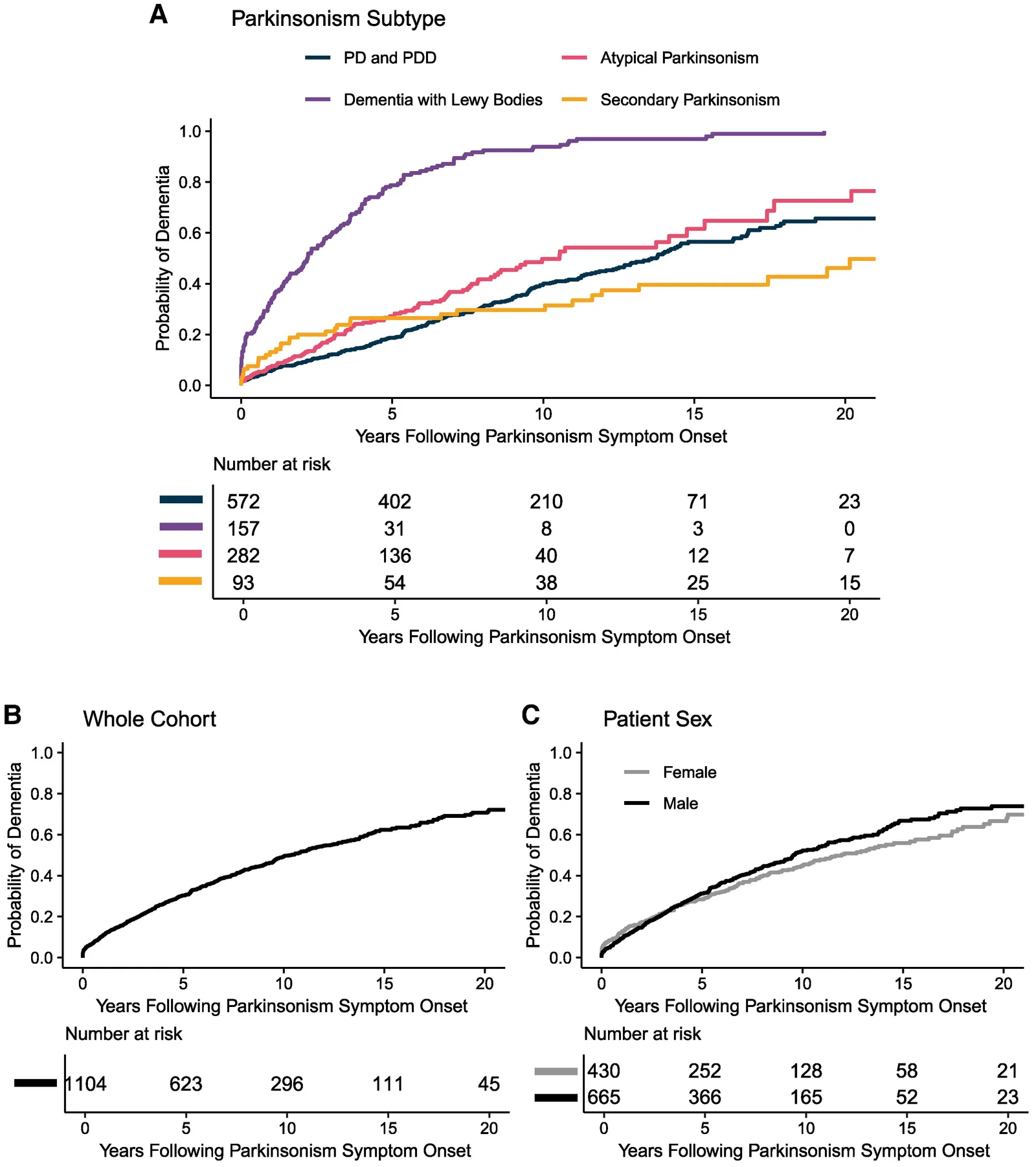
**Kaplan–Meier cumulative incidence of dementia following parkinsonism onset by diagnostic and demographic characteristics. A** Kaplan–Meier cumulative incidence of dementia by parkinsonism subtype (baseline numbers at risk: PD and PDD 572, dementia with Lewy bodies 157, atypical parkinsonism 282, secondary parkinsonism 93); **B** Kaplan–Meier cumulative incidence of dementia following parkinsonism symptom onset (baseline number at risk 1104); **C** Kaplan–Meier cumulative incidence of dementia in parkinsonism among males and females (baseline numbers at risk: female 430, male 665). PD, Parkinson’s disease; PDD, Parkinson’s disease with dementia.

At the most recent follow-up, 696 (63.0%) patients exhibited some degree of cognitive impairment, with 153 (13.9%) diagnosed with MCI and 543 (49.2%) diagnosed with dementia. Among those with dementia, 334 (61.5%) were males, 201 (37.0%) were females, and 8 (1.5%) did not disclose this information. In the whole cohort of parkinsonism, the cumulative incidence of dementia was 30.3% at 5 years after disease onset, 48.6% at 10 years, 61.4% at 15 years and 69.3% at 20 years. Among the PD/PDD cohort, 77 (13.5%) patients were diagnosed with MCI and 249 (43.5%) with dementia. In this group, the cumulative incidence of dementia was 18.9% at 5 years after disease onset, 40.3% at 10 years, 58.2% at 15 years and 67.8% at 20 years. Among the DLB cohort, 7 (4.5%) patients were diagnosed with MCI and 150 (95.5%) with dementia. In this group, the cumulative incidence of dementia was 77.9% at 5 years after disease onset, 92.8% at 10 years and 95.1% at 15 years. Among the atypical parkinsonism cohort, 54 (19.1%) patients were diagnosed with MCI and 109 (38.7%) with dementia. In this group, the cumulative incidence of dementia was 27.2% at 5 years after disease onset, 47.4% at 10 years, 58.3% at 15 years and 65.4% at 20 years. Among the secondary parkinsonism cohort, 15 (16.1%) patients were diagnosed with MCI and 35 (37.6%) with dementia. In this group, the cumulative incidence of dementia was 26.4% at 5 years after disease onset, 31.7% at 10 years, 40.1% at 15 years and 48.9% at 20 years (Fig. 1). A complete summary of cumulative incidence in the cohorts is provided in Table 2.

**Table 2:** Cumulative incidence of dementia following parkinsonism symptom onset

	Cumulative incidence (95% CI)			
Parkinsonism subtype	5 years	10 years^a^	15 years^b^	20 years^c^
All patients	30.3% (29.4, 31.1%)	48.6% (46.9, 50.4%)	61.4% (58.7, 64.3%)	69.3% (65.4, 73.4%)
PD/PDD	18.9% (18.3, 19.5%)	40.3% (38.4, 42.2%)	58.2% (54.8, 61.8%)	67.8% (62.8, 73.3%)
Dementia with Lewy bodies	77.9% (72.8, 83.4%)	92.8% (88.4, 97.4%)	95.1% (90.5, 99.9%)	—
Atypical parkinsonism	27.2% (25.7, 28.8%)	47.4% (43.5, 51.6%)	58.3% (51.3, 66.2%)	65.4% (55.1, 77.6%)
Secondary parkinsonism	26.4% (24.0, 29.0%)	31.7% (28.4, 35.5%)	40.1% (35.0, 46.1%)	48.9% (41.4, 57.7%)

PD, Parkinson’s disease; PDD, Parkinson’s disease with dementia.

^a^10-year cumulative incidence reported only for index years 1991–2015.

^b^15-year cumulative incidence reported only for index years 1991–2010.

^c^20-year cumulative incidence reported only for index years 1991–2005.

No significant difference in the incidence of cognitive impairment or dementia was found between PD/PDD and atypical parkinsonism or secondary parkinsonism. Conversely, as expected, an increased risk of cognitive impairment and dementia was found in patients with DLB compared to PD/PDD after adjusting for age and sex (HR = 4.41, 95% CI: 3.60–5.41, *P* < 0.001 and HR = 5.59, 95% CI: 4.50–6.95, *P* < 0.001), and after further adjusting for LASSO-selected risk factors (HR = 3.54, 95% CI: 2.89–4.39, *P* < 0.001 and HR = 4.73, 95% CI: 3.77–5.95, *P* < 0.001). A complete summary of the comparisons of patient risk for cognitive impairment or dementia between parkinsonism subtypes is provided in Table 3.

**Table 3:** Comparison of patient risk for cognitive impairment or dementia between parkinsonism subtypes

	Adjusted for age and sex		Further adjusted for LASSO-selected risk factors	
	Hazard ratio (95% CI)	*P*-value	Hazard ratio (95% CI)	*P*-value
Cognitive impairment or dementia				
PD/PDD	Reference	—	Reference	—
Dementia with Lewy bodies	4.41 (3.60, 5.41)	<0.001	3.54 (2.89, 4.39)	<0.001
Atypical parkinsonism	1.47 (1.21, 1.78)	<0.001	1.19 (0.97, 1.46)	0.099
Secondary parkinsonism	1.28 (0.93, 1.75)	0.13	1.24 (0.89, 1.71)	0.20
Dementia				
PD/PDD	Reference	—	Reference	—
Dementia with Lewy bodies	5.59 (4.50, 6.95)	<0.001	4.73 (3.77, 5.95)	<0.001
Atypical parkinsonism	1.17 (0.93, 1.48)	0.18	1.02 (0.81, 1.30)	0.85
Secondary parkinsonism	1.14 (0.78, 1.66)	0.49	1.11 (0.76, 1.61)	0.61
Dementia diagnosed by a neurologist^a^				
PD/PDD	Reference	—	Reference	—
Dementia with Lewy bodies	8.11 (6.21, 10.60)	<0.001	6.11 (4.54, 8.21)	<0.001
Atypical parkinsonism	0.93 (0.67, 1.29)	0.65	0.89 (0.64, 1.24)	0.49
Secondary parkinsonism	0.80 (0.46, 1.41)	0.45	0.84 (0.47, 1.47)	0.53

Statistical analysis was performed using Cox proportional hazards regression model using the PD/PDD group as reference.

PD, Parkinson’s disease; PDD, Parkinson’s disease with dementia.

^a^Patients with dementia diagnosed by a physician but not a neurologist were excluded from this analysis.

### Risk factors

After adjusting for sex and parkinsonism subtype, older age at disease onset was significantly associated with a higher risk of dementia, with a 35% increase per 5-year increment (HR = 1.35, 95% CI: 1.29–1.41, *P* < 0.001). This association was confirmed in a penalized model in both the whole cohort (HR = 1.32, 95% CI: 1.25–1.39, *P* < 0.001) and patients with PD/PDD only (HR = 1.25, 95% CI: 1.16–1.35, *P* < 0.001).

### Motor symptoms

In the whole cohort, bradykinesia (HR = 1.38, 95% CI: 1.11–1.71, *P* = 0.004) and impaired postural reflexes (HR = 1.51, 95% CI: 1.26–1.80, *P* < 0.001) were the cardinal motor symptoms independently associated with increased risk of dementia in univariable models after adjustments. Other secondary motor symptoms associated with dementia in univariable adjusted models were wide-based gait (HR = 1.46, 95% CI: 1.19–1.78, *P* < 0.001), dysarthria/dysphagia (HR = 1.45, 95% CI: 1.13–1.88, *P* = 0.003), en bloc movements (HR = 1.47, 95% CI: 1.13–1.90, *P* = 0.004) and axial rigidity (HR = 2.58, 95% CI: 1.56–4.25, *P* < 0.001). Notably, resting tremor was associated with a lower risk of dementia in univariable adjusted models (HR = 0.73, 95% CI: 0.60–0.88, *P* < 0.001). In the LASSO-penalized multivariable Cox regression model, a statistically significant association with dementia was confirmed for impaired postural reflexes (HR = 1.26, 95% CI: 1.05–1.51, *P* = 0.014). In PD/PDD patients only, no secondary motor symptoms were associated with dementia in the LASSO-penalized multivariable Cox regression model.

### Eye movement abnormalities

In the whole cohort, smooth pursuit alterations (HR = 1.82, 95% CI: 1.41–2.34, *P* < 0.001), reduced ocular range of motion (HR = 2.41, 95% CI: 1.78–3.26, *P* < 0.001) and saccades abnormalities (HR = 1.57, 95% CI: 1.10–2.23, *P* < 0.001) were the eye movement abnormalities associated with dementia in univariable adjusted models. In the LASSO-penalized multivariable Cox regression model, a statistically significant association with dementia was confirmed for smooth pursuit alterations (HR = 1.34, 95% CI: 1.04–1.74, *P* = 0.024) and reduced ocular range of motion (HR = 1.86, 95% CI: 1.37–2.54, *P* < 0.001).

In PD/PDD patients only, a statistically significant association with dementia in the LASSO-penalized multivariable Cox regression model was found for smooth pursuit alterations (HR = 1.68, 95% CI: 1.12–2.53, *P* = 0.012) and reduced ocular range of motion (HR = 4.49, 95% CI: 2.76–7.30, *P* < 0.001).

### Non-motor symptoms

In the whole cohort, constipation (HR = 1.50, 95% CI: 1.26–1.80, *P* < 0.001), orthostatic hypotension (HR = 1.56, 95% CI: 1.29–1.88, *P* < 0.001), erectile dysfunction (HR = 1.30, 95% CI: 1.04–1.64, *P* = 0.013) and RBD (HR = 1.75, 95% CI: 1.41–2.17, *P* < 0.001) were the non-motor symptoms associated with dementia in univariable adjusted models. In the LASSO-penalized multivariable Cox regression model, a statistically significant association with dementia was confirmed for constipation (HR = 1.24, 95% CI: 1.03–1.50, *P* = 0.022), orthostatic hypotension (HR = 1.30, 95% CI: 1.07–1.59, *P* = 0.009) and RBD (HR = 1.51, 95% CI: 1.21–1.89, *P* < 0.001).

In PD/PDD patients only, a statistically significant association with dementia in the LASSO-penalized multivariable Cox regression model was found for constipation (HR = 1.46, 95% CI: 1.11–1.93, *P* = 0.007) and orthostatic hypotension (HR = 1.78, 95% CI: 1.33–2.39, *P* < 0.001).

### Imaging abnormalities

In the whole cohort, both basal ganglia infarction (HR = 1.37, 95% CI: 1.07–1.75, *P* = 0.012) and hydrocephalus (HR = 1.72, 95% CI: 1.04–2.86, *P* = 0.034) were imaging abnormalities associated with dementia. In the LASSO-penalized multivariable Cox regression model, neither variable remained significant in the whole cohort or in patients with PD/PDD. A complete summary of dementia risk factor analyses in the whole cohort and patients with PD/PDD is provided in Table 4.

**Table 4:** Risk factors for dementia

	Univariable models		LASSO multivariable model^b^		PD/PDD only (*N* = 572)^b^	
Clinical features	Hazard ratio (95% CI)^a^	*P*-value	Hazard ratio (95% CI)^a^	*P*-value	Hazard ratio (95% CI)^a^	*P*-value
Age, per 5-year increase	1.35 (1.29, 1.41)	<0.001	1.32 (1.25, 1.39)	<0.001	1.25 (1.16, 1.35)	<0.001
Sex, male	1.13 (0.95, 1.34)	0.17	1.14 (0.95, 1.37)	0.16	1.14 (0.87, 1.50)	0.33
Cardinal motor symptoms						
Rest tremor	0.73 (0.60, 0.88)	<0.001	—	—	—	—
Bradykinesia	1.38 (1.11, 1.71)	0.004	—	—	—	—
Rigidity	1.13 (0.93, 1.37)	0.23	—	—	—	—
Impaired postural reflexes	1.51 (1.26, 1.80)	<0.001	1.26 (1.05, 1.51)	0.014	1.26 (0.96, 1.65)	0.090
Secondary motor symptoms						
Wide-based gait	1.46 (1.19, 1.78)	<0.001	1.13 (0.93, 1.39)	0.22	1.23 (0.89, 1.69)	0.21
Dysarthria/dysphagia	1.45 (1.13, 1.88)	0.003	—	—	—	—
En-bloc movements	1.47 (1.13, 1.90)	0.004	—	—	—	—
Focal dystonia	1.18 (0.79, 1.76)	0.41	—	—	—	—
Cerebellar symptoms	1.45 (0.95, 2.22)	0.083	—	—	—	—
Axial rigidity	2.58 (1.56, 4.25)	<0.001	—	—	—	—
Eye movement abnormalities						
Smooth pursuit alterations	1.82 (1.41, 2.34)	<0.001	1.34 (1.04, 1.74)	0.024	1.68 (1.12, 2.53)	0.012
Reduced ocular range of motion	2.41 (1.78, 3.26)	<0.001	1.86 (1.37, 2.54)	< 0.001	4.49 (2.76, 7.30)	< 0.001
Saccade abnormalities	1.57 (1.10, 2.23)	<0.001	—	—	—	—
Non-motor symptoms						
Constipation	1.50 (1.26, 1.80)	0.001	1.24 (1.03, 1.50)	0.022	1.46 (1.11, 1.93)	0.007
Orthostatic hypotension	1.56 (1.29, 1.88)	<0.001	1.30 (1.07, 1.59)	0.009	1.78 (1.33, 2.39)	< 0.001
Erectile dysfunction	1.30 (1.04, 1.64)	0.013	—	—	—	—
REM sleep behavior disorder	1.75 (1.41, 2.17)	< 0.001	1.51 (1.21, 1.89)	< 0.001	0.86 (0.61, 1.21)	0.38
Neurogenic bladder	1.26 (0.95, 1.68)	0.11	—	—	—	—
Imaging abnormalities						
Basal ganglia infarction	1.37 (1.07, 1.75)	0.012	—	—	—	—
Hydrocephalus	1.72 (1.04, 2.86)	0.034	—	—	—	—

Statistical analysis was performed using Cox proportional hazards regression. For multivariable models, variable selection was performed using an LASSO penalization.

LASSO, least absolute shrinkage and selection operator; PD, Parkinson’s disease; PDD, Parkinson’s disease with dementia; REM, rapid eye movement.

^a^Hazard ratios were adjusted for patient age at parkinsonism onset, sex and parkinsonism subtype.

^b^Risk factors not included after LASSO penalty are denoted with —.

The same analysis on motor symptoms, non-motor symptoms, and imaging abnormalities was performed for dementia and MCI together. In addition to the variables identified in the dementia-only models, prominent cerebellar symptoms and neurogenic bladder were associated with cognitive impairment in univariable adjusted models. In the LASSO-penalized multivariable Cox regression model, neither variable remained significant. A complete summary of MCI/dementia risk factor analyses in the whole cohort and in patients with PD/PDD is provided in Table 5.

**Table 5:** Risk factors for mild cognitive impairment or dementia

	Univariable models		LASSO multivariable model^b^		PD/PDD only (*N* = 572)^b^	
Clinical features	Hazard ratio (95% CI)^a^	*P*-value	Hazard ratio (95% CI)^a^	*P*-value	Hazard ratio (95% CI)^a^	*P*-value
Age, per 5-year increase	1.32 (1.27, 1.38)	<0.001	1.28 (1.23, 1.34)	<0.001	1.26 (1.17, 1.34)	<0.001
Sex, male	1.21 (1.04, 1.41)	0.014	1.20 (1.02, 1.41)	0.030	1.23 (0.97, 1.56)	0.092
Cardinal motor symptoms						
Rest tremor	0.77 (0.65, 0.91)	<0.001	—	—	—	—
Bradykinesia	1.28 (1.06, 1.54)	0.010	—	—	—	—
Rigidity	1.17 (0.98, 1.39)	0.077	—	—	—	—
Impaired postural reflexes	1.48 (1.27, 1.74)	<0.001	1.26 (1.07, 1.48)	0.005	1.13 (0.89, 1.43)	0.31
Secondary motor symptoms						
Wide-based gait	1.54 (1.29, 1.83)	<0.001	1.23 (1.03, 1.46)	0.025	1.28 (0.97, 1.68)	0.084
Dysarthria/dysphagia	1.45 (1.13, 1.88)	0.004	—	—	—	—
En bloc movements	1.53 (1.22, 1.93)	<0.001	—	—	—	—
Focal dystonia	1.13 (0.80, 1.61)	0.49	—	—	—	—
Cerebellar symptoms	1.71 (1.21, 2.41)	0.002	—	—	—	—
Axial rigidity	2.43 (1.55, 3.81)	<0.001	—	—	—	—
Eye movement abnormalities						
Smooth pursuit alterations	1.72 (1.38, 2.16)	<0.001	1.30 (1.03, 1.63)	0.028	1.55 (1.07, 2.24)	0.020
Reduced ocular range of motion	2.47 (1.89, 3.24)	<0.001	1.61 (1.22, 2.13)	< 0.001	3.71 (2.34, 5.87)	<0.001
Saccade abnormalities	1.80 (1.34, 2.42)	<0.001	—	—	—	—
Non-motor symptoms						
Constipation	1.47 (1.25, 1.72)	<0.001	1.24 (1.05, 1.46)	0.010	1.40 (1.10, 1.78)	0.006
Orthostatic hypotension	1.64 (1.39, 1.93)	<0.001	1.39 (1.17, 1.65)	< 0.001	1.71 (1.33, 2.19)	<0.001
Erectile dysfunction	1.17 (0.96, 1.44)	0.13	—	—	—	—
REM sleep behavior disorder	1.77 (1.46, 2.15)	<0.001	1.48 (1.21, 1.80)	< 0.001	1.04 (0.77, 1.39)	0.81
Neurogenic bladder	1.44 (1.13, 1.74)	0.002	—	—	—	—
Imaging abnormalities						
Basal ganglia infarction	1.40 (1.13, 1.74)	0.002	1.19 (0.96, 1.49)	0.12	1.58 (1.14, 2.20)	0.007
Hydrocephalus	2.45 (1.61, 3.74)	<0.001	—	—	—	—

Statistical analysis was performed using Cox proportional hazards regression. For multivariable models, variable selection was performed using an LASSO penalization.

LASSO, least absolute shrinkage and selection operator; PD, Parkinson’s disease; PDD, Parkinson’s disease with dementia; REM, rapid eye movement.

^a^Hazard ratios were adjusted for patient age at parkinsonism onset, sex and parkinsonism subtype.

^b^Risk factors not included after LASSO penalty are denoted with —.

## Discussion

### Demographics

Analyzing data from a large population-based cohort, we found a 63.0% cumulative probability of developing any cognitive impairment and a 49.2% cumulative probability of developing dementia after 5–35 years of follow-up. The cumulative incidence of dementia showed a steady increase during follow-up, rising from approximately 30% at 5 years to 70% at 20 years. Particularly, patients with PD/PDD presented an 18.9% cumulative incidence of dementia at 5 years, which increased to 40.3% at 10 years, 58.2% at 15 years and 67.8% at 20 years. These data confirm the results of the most recent studies on dementia in parkinsonism.^13,30-32^ Considering that 19.2% of patients were not followed until death, we acknowledge that the observed cumulative incidence may increase as additional follow-up data become available for surviving individuals.

As expected, the majority of patients with dementia were men (61.5%). However, the sex difference over such a long follow-up was less pronounced than previously reported.^13,33^ Notably, this difference was greater when considering both MCI and dementia. Age at onset was also a strong predictor of cognitive impairment in this cohort, consistent with previous literature, which reported a higher prevalence of dementia among older individuals in both parkinsonism and PD.^13,32,34^

### Motor symptoms

**Figure 2:**
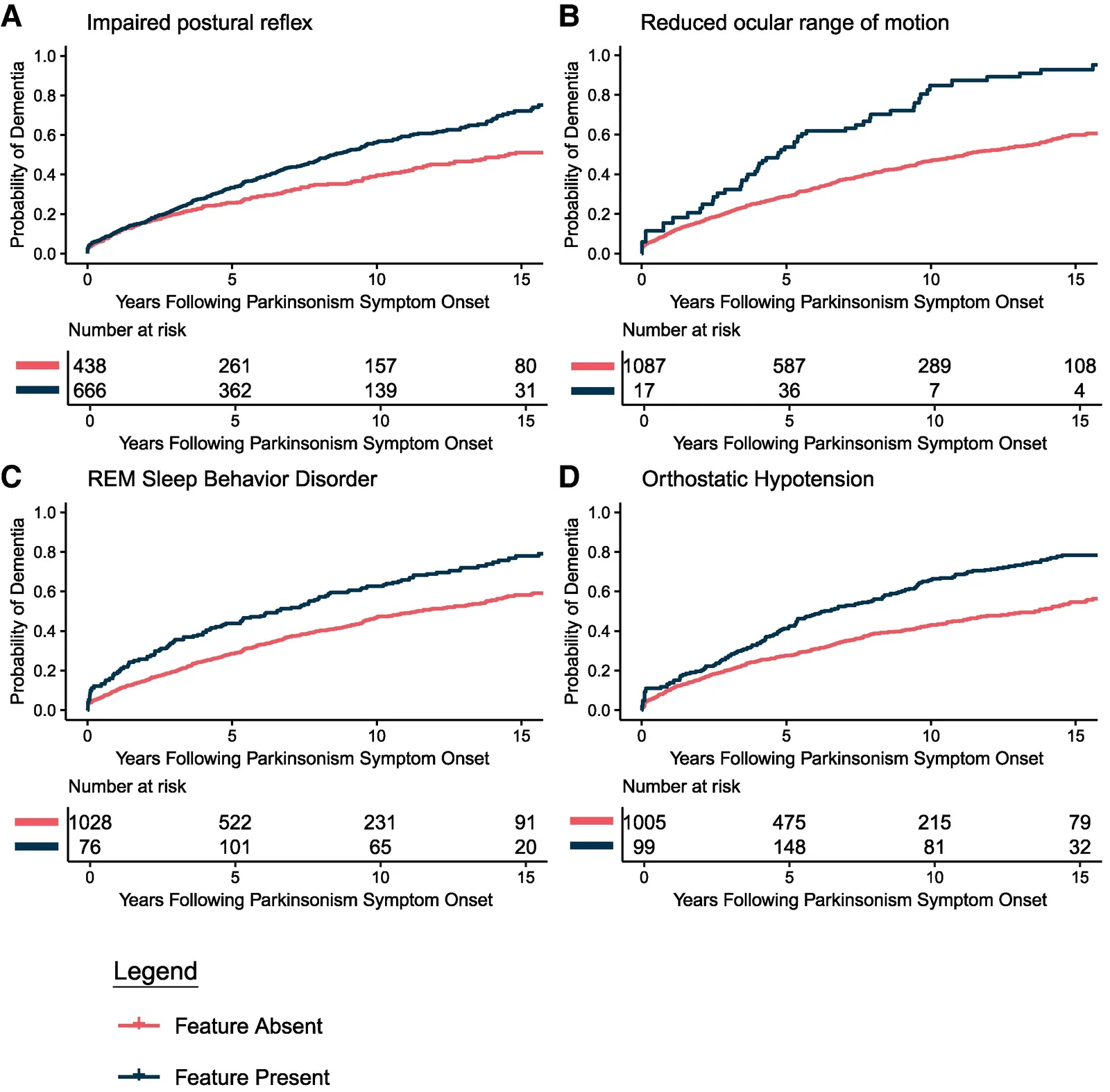
**Kaplan–Meier cumulative incidence of dementia following parkinsonism onset by symptom status in the whole cohort. A** Kaplan–Meier cumulative incidence of dementia by impaired postural reflexes (baseline numbers at risk: feature absent 438, feature present 666); **B** Kaplan–Meier cumulative incidence of dementia by reduced ocular range of motion (baseline numbers at risk: feature absent 1087, feature present 17); **C** Kaplan–Meier cumulative incidence of dementia by REM sleep behaviour disorder (baseline numbers at risk: feature absent 1028, feature present 76); **D** Kaplan–Meier cumulative incidence of dementia by orthostatic hypotension (baseline numbers at risk: feature absent 1005, feature present 99).

Our results identified several clinical features as risk factors for dementia in the whole cohort across parkinsonism subtypes and specifically in patients with PD/PDD (Fig. 2). Particularly, bradykinesia and impaired postural reflexes were associated with dementia, with the latter remaining significant even when accounting for collinearity with other variables in the LASSO-penalized model in the whole cohort. The postural instability-gait difficulty PD subtype has been associated with increased risk of dementia, and this finding is supported by imaging analysis demonstrating greater grey matter atrophy in several brain regions.^35-38^ Specifically, reduced volume has been observed in the pre-supplementary motor area and postcentral gyri, with decreased functional connectivity between cortical and subcortical motor-planning networks.^35^ Frontal regions closely linked with cognition and strongly implicated in executive tasks and decision-making have also demonstrated a greater atrophy in these patients.^39^ Importantly, these postural and gait features appear to be partially independent of dopaminergic deficits and have been strongly linked to cholinergic abnormalities, as shown in both post-mortem neuropathology and in vivo imaging studies.^38,40,41^ Particularly, PET studies and animal models suggested that patients with PD characterized by isolated falls or freezing of gait exhibit cholinergic deficits within components of the attentional-motor interface network, involving striatal cholinergic interneurons responsible for integrating attention and motor control.^38,42^ This overlap of affected cholinergic pathways involved in motor control and cognition may explain the increased risk of dementia observed in this symptomatic subtype.^40,43^ Finally, cognitive decline may also be the primary driver of postural imbalance, as impaired executive function has been found to increase the risk of falls, highlighting the interdependence of these aspects.^44,45^

The bradykinetic-akinetic PD phenotype has also been associated with a poorer prognosis and increased risk of dementia.^8,46,47^ This phenotype has been mainly attributed to alterations in the anatomy and function of the basal ganglia motor loops.^48,49^ However, bradykinesia/akinesia appears to have a pathophysiology that extends beyond dopaminergic pathways, involving alterations in connectivity and volume within the frontoparietal network, as well as the cerebello-thalamo-cortical and mesolimbic-striatal pathways.^48,50,51^ In our cohort, the association between dementia and bradykinesia/akinesia was further supported by the statistical significance of en bloc movements as a predictive risk factor in patients with PD/PDD and across all parkinsonism subtypes. However, this association was not confirmed in the penalized analysis, likely because it was partially mediated by other variables, including bradykinesia. Notably, rigidity was not associated with dementia in either parkinsonism or the PD/PDD group. However, prominent or isolated axial rigidity showed a significant role as a predictive factor, once again suggesting a correlation between dementia and akinetic-rigid phenotype, particularly when rigidity affects the trunk and proximal musculature rather than the extremities, as suggested by other retrospective and prospective studies.^52,53^ Resting tremor showed a slightly reduced risk of cognitive impairment in univariable adjusted models, suggesting a possible milder progression in patients with resting-tremor-predominant Parkinson’s disease.

Secondary motor symptoms associated with dementia included dysarthria/dysphagia and wide-based gait. Dysarthria and dysphagia in PD have been associated with both basal ganglia and cortical sensorimotor integration, and alterations of these circuits have also been implicated in multiple cognitive domains and in the development of dementia-related processes.^54^ Particularly, functional MRI analyses have revealed a primary dysfunction of the mesial fronto-striatal network in PD patients with hypokinetic dysarthria, suggesting crucial cortical involvement in the development of this symptom.^55^ Beyond hypokinetic dysarthria, spastic dysarthria has also been frequently observed in atypical parkinsonism such as PSP, which is characterized by a high prevalence of cognitive impairment.^56,57^ PD patients with dysphagia have also demonstrated a marked reduction in task-related cortical activation on functional imaging compared with non-dysphagic patients, suggesting involvement of non-dopaminergic networks as well.^58^ Wide-based gait is another characteristic feature of atypical parkinsonism, observed especially in PSP, cerebellar variant of MSA, and VP. Consistent with these observations, this feature was associated with dementia in the whole cohort but not in the PD/PDD group, which typically exhibits a narrow-based gait impairment.^59-61^ This characteristic narrow-based gait in PD appears to be primarily linked to a reduced trunk motion and increased trunk rigidity, which has no impact on sideways stability.^62^

### Eye movement abnormalities

Smooth pursuit alterations and reduced ocular range of motion were strongly associated with cognitive decline in this study, both in the whole cohort and in patients with PD/PDD only. Smooth pursuit tracking abnormalities manifest as reduced gain or speed of saccadic pursuit, and are commonly seen in PD with a frequency of up to 75%.^63^ Previous studies have suggested that these features are likely correlated with both dopaminergic and non-dopaminergic pathways.^64^ Specifically, these dysfunctional eye movements appear to be primarily related to cortical executive deficits attributable to higher-order functional networks.^65^ Mild reduced ocular range of motion, typically responsive to dopaminergic treatment, has been described in PD cohorts.^66,67^ These features involve a network comprising the frontal eye fields, parietal cortex, superior colliculus, and cerebellum, circuits closely linked to cognitive impairment as well.^65,68,69^ This is especially relevant in PSP, where upward gaze limitation is a well-established diagnostic criterion strongly associated with disease severity.^70,71^ Additionally, recent studies in cohorts of patients with DLB have described diminished visual exploration and altered eye movement patterns.^72^ Altered saccades include hypometria, difficulties in shifting gaze and increased latency and are linked to both motor and cognitive impairment.^69,73^ Specifically, in patients with PD, altered saccades have demonstrated predictive power for cognitive decline across global cognition, executive function, attention, and memory domains.^73^ However, the substantia nigra pars reticulata, typically affected in the initial stages of the disease, has been shown to play a significant role in the generation of voluntary saccades.^64,74^ In line with these findings, saccade abnormalities showed a weaker significant association with dementia in univariable adjusted analysis, and no association after penalized adjustment.

### Non-motor symptoms

Among non-motor features, autonomic dysfunctions such as orthostatic hypotension and constipation were found to be associated with dementia across parkinsonism subtypes and in the PD/PDD group. These symptoms are common in parkinsonism, particularly in MSA, and may even precede the onset of motor symptoms.^75,76^ The deposition of alpha-synuclein in autonomic neurons is considered a key factor contributing to autonomic dysfunction in parkinsonism.^77^ Specifically, post-mortem studies have reported alpha-synuclein accumulation in the hypothalamus, insula, and dorsal motor nucleus of the vagus.^78,79^ Additionally, functional imaging studies in PD patients with autonomic dysfunction have reported a poorer performance in frontal-executive tasks and a deterioration of functional and structural connectivity in both frontal and posterior cortical regions.^80^ A correlation between the severity of autonomic dysfunction and reduced white matter connectivity in fronto-subcortical and posterior cortical regions was also observed.^80^ These findings may reflect more advanced Lewy body pathology, a well-established pathogenic substrate of cognitive impairment.^80^ Orthostatic hypotension has been specifically linked to recurrent cerebral hypoperfusion.^81-83^ These transient and repeated bouts of cerebral hypoperfusion may induce chronic hypoxic damage and activate specific molecular pathways, leading to neurodegeneration.^81-83^ This additional mechanism may explain the stronger association between orthostatic hypotension and dementia observed in our cohort. Gastrointestinal autonomic symptoms, particularly constipation, have also been associated with poorer performance on verbal learning, visuospatial learning, attention, and memory in patients with PD.^84,85^ A possible explanation is that these symptoms may reflect longstanding gut microbiota dysregulation, leading to increased intestinal permeability, oxidative stress, and localized inflammation, which may subsequently influence cerebral function through the gut–brain axis; however, the underlying mechanisms remain incompletely understood.^77,84^

RBD is another clinical feature that has shown an association with dementia across parkinsonism, even after adjustment in the penalized model analysis. This disorder is known to be predictive of faster cognitive decline and has been related to dysfunction across neuronal networks, including the hypothalamus, thalamus, amygdala, anterior cingulate cortex, posterior cingulate cortex, and hippocampus.^86-88^ Specifically, within the Lewy body spectrum disorders, RBD has been shown to arise from degeneration of pontomedullary cholinergic pathways caused by Lewy bodies accumulation.^89,90^ Neuronal loss in the cholinergic pedunculopontine nucleus and disrupted resting-state functional connectivity of the basal nucleus of Meynert are also believed to contribute to RBD pathophysiology.^91,92^ As described earlier, dysfunction of the cholinergic network is closely linked to cognitive impairment and dementia, thus explaining its association with RBD.^40^ Moreover, several clinical studies have reported that patients with RBD tend to perform poorly across multiple cognitive domains, including global cognition, long-term verbal recall and recognition, language and visuospatial ability.^91^ Notably, in contrast with the majority of the literature, when considering patients with PD/PDD only, this association was not statistically significant. A possible explanation for this finding may be that patients with prodromal RBD develop some degree of cognitive impairment prior to the onset of motor symptoms, and therefore, they have been excluded from the present analysis. Another explanation may be an incomplete ascertainment of RBD history among patients with PD/PDD compared to other parkinsonism more frequently associated with RBD such as DLB, partially due to the retrospective design of the study.^90,93^ The substantial body of evidence supporting cholinergic system impairment in patients with RBD warrants a cautious interpretation of this finding and underscores the need for further research into the role of this feature as a potential predictor of dementia.

### Imaging abnormalities

Neuroimaging findings, including basal ganglia infarction and hydrocephalus, were associated with dementia in univariable adjusted models. The associations between these abnormalities and cognitive decline are well-established: patients with cerebrovascular events have an increased risk of developing vascular dementia^94,95^; cognitive impairment represents a core diagnostic criterion of the Hakim-Adams triad in normal pressure hydrocephalus.^96^ Since pseudoparkinsonism aetiologies, such as normal pressure hydrocephalus, were excluded by the selection criteria, these patients were either initially misdiagnosed or may have developed such features as comorbidities in the presence of a parkinsonian neurodegenerative process.

In our cohort, 63.0% of patients with parkinsonism developed some degree of cognitive impairment after up to 35 years of follow-up. Particularly, patients with PD/PDD had a cumulative incidence of dementia of 18.9% at 5 years, 40.3% at 10 years, 58.2% at 15 years and 67.8% at 20 years, consistent with recent evidence suggesting that dementia is not inevitable in PD even after a prolonged follow-up. As expected, older age was a predictor of cognitive impairment, and most affected individuals were males. Analysis of predictive factors confirmed a strong association between dementia and akinetic-rigid-predominant or postural instability-gait difficulty subtypes in both PD/PDD and the whole parkinsonism cohort, supported by additional significant risk factors such as axial rigidity, en bloc movements and dysarthria/dysphagia. Eye movement abnormalities, particularly reduced ocular range of motion and smooth pursuit alterations, were among the strongest risk factors for dementia in PD/PDD and parkinsonism patients, supporting the involvement of distributed subcortical and cortical networks. Similarly, autonomic features, including orthostatic hypotension and constipation, were significant predictors, suggesting early central autonomic involvement in the neurodegenerative process. Finally, RBD was linked to dementia across all parkinsonism subtypes, reflecting involvement of non-dopaminergic pathways. Notably, this association was not significant when analysing patients with PD/PDD only, a finding that warrants cautious interpretation.

Further research is needed to confirm these findings and to elucidate the underlying mechanisms, including neurotransmitter alterations, regional central nervous system involvement and network disruption that contribute to cognitive decline across parkinsonism subtypes. Nonetheless, our results highlight clinical features that may indicate a worse prognosis and an increased risk of cognitive impairment, thereby informing clinical management, and supporting earlier therapeutic interventions.

### Strengths and limitations

The results of this analysis are strengthened by several factors. First, all incident cases of parkinsonism from a defined population over a specific time window were included, thereby minimizing referral bias compared to previous studies that recruited patients from hospitals or communities, which may have resulted in the inclusion of more advanced or atypical cases. Second, analysis was conducted on a large cohort (*n* = 1104) and included multiple parkinsonism subtypes (PD, PDD, DLB, MSA, PSP, VP, DIP, SP and unspecified parkinsonism), resulting in a broader and more representative sample than many previous studies. Third, each parkinsonism diagnosis was validated by a movement disorder specialist. Fourth, all dementia diagnoses were supported by either formal neuropsychological testing or brief cognitive screening and then retrospectively confirmed by a physician. Finally, the study covered a 30-year enrolment period (1991–2020) with an observation period up to 35 years, and follow-up extending through January 2025.

However, some limitations should be acknowledged. First, a group of unaffected controls was not included, thereby limiting our ability to draw conclusions about the general population. Second, the sample size of certain atypical parkinsonism syndromes was relatively small. Third, 213 cases were classified as UP due to atypical presentations that did not meet established diagnostic criteria, likely introducing diagnostic heterogeneity within the atypical group and limiting the possibility of performing a dedicated analysis of this group. Fourth, clinical variability within the whole cohort and within diagnostic groups may reduce interpretability of findings across specific parkinsonism subtypes; however, this grouping approach was selected to reflect clinically and pathologically meaningful distinctions. Fifth, the retrospective longitudinal nature of the study is inherently associated with misclassification bias, potentially leading to underestimation of clinical features. Additionally, due to this study design, patients could not undergo a consistent and standardized cognitive evaluation. Finally, not all patients were followed until death, thereby limiting the completeness of estimates of dementia cumulative incidence.

## Data Availability

Data not provided in the article may be shared anonymized at the request of any qualified investigator. Data are not publicly available due to privacy or ethical restrictions. The code used for the analyses in this study is available from the corresponding author upon reasonable request and will be shared in accordance with institutional policies.

## References

[1] Poewe W, Seppi K, Tanner CM, et al Parkinson disease. Nat Rev Dis Primers. 2017;3:17013. 28332488 10.1038/nrdp.2017.13

[2] Postuma RB, Berg D, Stern M, et al MDS clinical diagnostic criteria for Parkinson's disease. Mov Disord. 2015;30(12):1591–1601. 26474316 10.1002/mds.26424

[3] Kalia LV, Lang AE. Parkinson's disease. Lancet. 2015;386(9996):896–912. 25904081 10.1016/S0140-6736(14)61393-3

[4] Pausch C, Schomburg R, Wagenpfeil S, et al Neuropsychological impairment in prodromal Parkinson's disease. J Neurol Sci. 2016;371:117–120. 27871431 10.1016/j.jns.2016.10.007

[5] Roheger M, Kalbe E, Liepelt-Scarfone I. Progression of cognitive decline in Parkinson's disease. J Parkinsons Dis. 2018;8(2):183–193. 29914040 10.3233/JPD-181306 PMC6004891

[6] Anang JB, Gagnon JF, Bertrand JA, et al Predictors of dementia in Parkinson disease: A prospective cohort study. Neurology. 2014;83(14):1253–1260. 25171928 10.1212/WNL.0000000000000842 PMC4180482

[7] Svenningsson P, Westman E, Ballard C, Aarsland D. Cognitive impairment in patients with Parkinson's disease: Diagnosis, biomarkers, and treatment. Lancet Neurol. 2012;11(8):697–707. 22814541 10.1016/S1474-4422(12)70152-7

[8] Forsaa EB, Larsen JP, Wentzel-Larsen T, Alves G. What predicts mortality in Parkinson disease?: A prospective population-based long-term study. Neurology. 2010;75(14):1270–1276. 20921512 10.1212/WNL.0b013e3181f61311

[9] Aarsland D, Creese B, Politis M, et al Cognitive decline in Parkinson disease. Nat Rev Neurol. 2017;13(4):217–231. 28257128 10.1038/nrneurol.2017.27 PMC5643027

[10] Litvan I, Aarsland D, Adler CH, et al MDS Task Force on mild cognitive impairment in Parkinson's disease: Critical review of PD-MCI. Mov Disord. 2011;26(10):1814–1824. 21661055 10.1002/mds.23823 PMC3181006

[11] Lawson RA, Yarnall AJ, Duncan GW, et al Stability of mild cognitive impairment in newly diagnosed Parkinson's disease. J Neurol Neurosurg Psychiatry. 2017;88(8):648–652. 28250029 10.1136/jnnp-2016-315099 PMC5537517

[12] Santangelo G, Vitale C, Picillo M, et al Mild cognitive impairment in newly diagnosed Parkinson's disease: A longitudinal prospective study. Parkinsonism Relat Disord. 2015;21(10):1219–1226. 26321021 10.1016/j.parkreldis.2015.08.024

[13] Gallagher J, Gochanour C, Caspell-Garcia C, et al Long-term dementia risk in Parkinson disease. Neurology. 2024;103(5):e209699. 39110916 10.1212/WNL.0000000000209699 PMC11318527

[14] Gibson LL, Weintraub D, Lemmen R, et al Risk of dementia in Parkinson's disease: A systematic review and meta-analysis. Mov Disord. 2024;39(10):1697–1709. 39036849 10.1002/mds.29918

[15] Koros C, Stefanis L, Scarmeas N. Parkinsonism and dementia. J Neurol Sci. 2022;433:120015. 34642023 10.1016/j.jns.2021.120015

[16] Davis AA, Racette B. Parkinson disease and cognitive impairment: Five new things. Neurol Clin Pract. 2016;6(5):452–458. 27847686 10.1212/CPJ.0000000000000285 PMC5100708

[17] Sousa-Fraguas MC, Rodriguez-Fuentes G, Conejo NM. Frailty and cognitive impairment in Parkinson's disease: A systematic review. Neurol Sci. 2022;43(12):6693–6706. 36056182 10.1007/s10072-022-06347-7

[18] Irwin DJ, White MT, Toledo JB, et al Neuropathologic substrates of Parkinson disease dementia. Ann Neurol. 2012;72(4):587–598. 23037886 10.1002/ana.23659 PMC3484250

[19] Emre M, Aarsland D, Brown R, et al Clinical diagnostic criteria for dementia associated with Parkinson's disease. Mov Disord. 2007;22(12):1689–1707; quiz 1837. 17542011 10.1002/mds.21507

[20] Guo Y, Liu FT, Hou XH, et al Predictors of cognitive impairment in Parkinson's disease: A systematic review and meta-analysis of prospective cohort studies. J Neurol. 2021;268(8):2713–2722. 32162063 10.1007/s00415-020-09757-9

[21] Hindle JV, Martyr A, Clare L. Cognitive reserve in Parkinson's disease: A systematic review and meta-analysis. Parkinsonism Relat Disord. 2014;20(1):1–7. 24034887 10.1016/j.parkreldis.2013.08.010

[22] Savica R, Grossardt BR, Bower JH, et al Survival and causes of death among people with clinically diagnosed synucleinopathies with parkinsonism: A population-based study. JAMA Neurol. 2017;74(7):839–846. 28505261 10.1001/jamaneurol.2017.0603 PMC5647647

[23] St Sauver JL, Grossardt BR, Yawn BP, Melton LJ 3rd, Rocca WA. Use of a medical records linkage system to enumerate a dynamic population over time: The Rochester epidemiology project. Am J Epidemiol. 2011;173(9):1059–1068. 21430193 10.1093/aje/kwq482 PMC3105274

[24] Poewe W, Gauthier S, Aarsland D, et al Diagnosis and management of Parkinson's disease dementia. Int J Clin Pract. 2008;62(10):1581–1587. 18822028 10.1111/j.1742-1241.2008.01869.x PMC2658001

[25] McKeith IG, Boeve BF, Dickson DW, et al Diagnosis and management of dementia with Lewy bodies: Fourth consensus report of the DLB consortium. Neurology. 2017;89(1):88–100. 28592453 10.1212/WNL.0000000000004058 PMC5496518

[26] Höglinger GU, Respondek G, Stamelou M, et al Clinical diagnosis of progressive supranuclear palsy: The movement disorder society criteria. Mov Disord. 2017;32(6):853–864. 28467028 10.1002/mds.26987 PMC5516529

[27] Camerucci E, Stang CD, Hajeb M, et al Early-onset parkinsonism and early-onset Parkinson's disease: A population-based study (2010–2015). J Parkinsons Dis. 2021;11(3):1197–1207. 33720851 10.3233/JPD-202464 PMC8355040

[28] Savica R, Grossardt BR, Bower JH, Ahlskog JE, Mielke MM, Rocca WA. Incidence and time trends of drug-induced parkinsonism: A 30-year population-based study. Mov Disord. 2017;32(2):227–234. 27779780 10.1002/mds.26839 PMC5318251

[29] Bower JH, Maraganore DM, McDonnell SK, Rocca WA. Incidence and distribution of parkinsonism in Olmsted County, Minnesota, 1976-1990. Neurology. 1999;52(6):1214–1220. 10214746 10.1212/wnl.52.6.1214

[30] Hely MA, Reid WG, Adena MA, Halliday GM, Morris JG. The Sydney multicenter study of Parkinson's disease: The inevitability of dementia at 20 years. Mov Disord. 2008;23(6):837–844. 18307261 10.1002/mds.21956

[31] Szwedo AA, Dalen I, Pedersen KF, et al GBA and APOE impact cognitive decline in Parkinson's disease: A 10-year population-based study. Mov Disord. 2022;37(5):1016–1027. 35106798 10.1002/mds.28932 PMC9362732

[32] Williams-Gray CH, Mason SL, Evans JR, et al The CamPaIGN study of Parkinson's disease: 10-year outlook in an incident population-based cohort. J Neurol Neurosurg Psychiatry. 2013;84(11):1258–1264. 23781007 10.1136/jnnp-2013-305277

[33] Cereda E, Cilia R, Klersy C, et al Dementia in Parkinson's disease: Is male gender a risk factor? Parkinsonism Relat Disord. 2016;26:67–72. 26952697 10.1016/j.parkreldis.2016.02.024

[34] Schrag A, Siddiqui UF, Anastasiou Z, Weintraub D, Schott JM. Clinical variables and biomarkers in prediction of cognitive impairment in patients with newly diagnosed Parkinson's disease: A cohort study. Lancet Neurol. 2017;16(1):66–75. 27866858 10.1016/S1474-4422(16)30328-3 PMC5377592

[35] Rosenberg-Katz K, Herman T, Jacob Y, Giladi N, Hendler T, Hausdorff JM. Gray matter atrophy distinguishes between Parkinson disease motor subtypes. Neurology. 2013;80(16):1476–1484. 23516323 10.1212/WNL.0b013e31828cfaa4 PMC3662357

[36] Alves G, Larsen JP, Emre M, Wentzel-Larsen T, Aarsland D. Changes in motor subtype and risk for incident dementia in Parkinson's disease. Mov Disord. 2006;21(8):1123–1130. 16637023 10.1002/mds.20897

[37] Yarnall A, Rochester L, Burn DJ. The interplay of cholinergic function, attention, and falls in Parkinson's disease. Mov Disord. 2011;26(14):2496–2503. 21898597 10.1002/mds.23932

[38] Bohnen NI, Yarnall AJ, Weil RS, et al Cholinergic system changes in Parkinson's disease: Emerging therapeutic approaches. Lancet Neurol. 2022;21(4):381–392. 35131038 10.1016/S1474-4422(21)00377-X PMC8985079

[39] Kirschner H, Ullsperger M. The medial frontal cortex, performance monitoring, cognitive control, and decision making. In: Grafman JH, ed. Encyclopedia of the human brain (second edition). Elsevier; 2025:112–126.

[40] Bohnen NI, Kanel P, Müller M. Molecular imaging of the cholinergic system in Parkinson's disease. Int Rev Neurobiol. 2018;141:211–250. 30314597 10.1016/bs.irn.2018.07.027 PMC6218162

[41] Karachi C, Grabli D, Bernard FA, et al Cholinergic mesencephalic neurons are involved in gait and postural disorders in Parkinson disease. J Clin Invest. 2010;120(8):2745–2754. 20628197 10.1172/JCI42642 PMC2912198

[42] Sarter M, Avila C, Kucinski A, Donovan E. Make a left turn: Cortico-striatal circuitry mediating the attentional control of complex movements. Mov Disord. 2021;36(3):535–546. 33615556 10.1002/mds.28532 PMC8938956

[43] Wu C, Wu H, Zhou C, et al Cholinergic basal forebrain system degeneration underlies postural instability/gait difficulty and attention impairment in Parkinson's disease. Eur J Neurol. 2024;31(2):e16108. 37877681 10.1111/ene.16108 PMC11235900

[44] Atkinson HH, Rosano C, Simonsick EM, et al Cognitive function, gait speed decline, and comorbidities: The health, aging and body composition study. J Gerontol A Biol Sci Med Sci. 2007;62(8):844–850. 17702875 10.1093/gerona/62.8.844

[45] Mirelman A, Herman T, Brozgol M, et al Executive function and falls in older adults: New findings from a five-year prospective study link fall risk to cognition. PLoS One. 2012;7(6):e40297. 22768271 10.1371/journal.pone.0040297 PMC3386974

[46] Williams-Gray CH, Foltynie T, Brayne CE, Robbins TW, Barker RA. Evolution of cognitive dysfunction in an incident Parkinson's disease cohort. Brain. 2007;130(Pt 7):1787–1798. 17535834 10.1093/brain/awm111

[47] Wojtala J, Heber IA, Neuser P, et al Cognitive decline in Parkinson's disease: The impact of the motor phenotype on cognition. J Neurol Neurosurg Psychiatry. 2019;90(2):171–179. 30297519 10.1136/jnnp-2018-319008

[48] Rodriguez-Oroz MC, Jahanshahi M, Krack P, et al Initial clinical manifestations of Parkinson's disease: Features and pathophysiological mechanisms. Lancet Neurol. 2009;8(12):1128–1139. 19909911 10.1016/S1474-4422(09)70293-5

[49] Wichmann T, DeLong MR, Guridi J, Obeso JA. Milestones in research on the pathophysiology of Parkinson's disease. Mov Disord. 2011;26(6):1032–1041. 21626548 10.1002/mds.23695 PMC4272856

[50] Kann SJ, Chang C, Manza P, Leung HC. Akinetic rigid symptoms are associated with decline in a cortical motor network in Parkinson's disease. NPJ Parkinsons Dis. 2020;6:19. 32885038 10.1038/s41531-020-00120-3 PMC7445297

[51] Zhang J, Wei L, Hu X, et al Akinetic-rigid and tremor-dominant Parkinson's disease patients show different patterns of intrinsic brain activity. Parkinsonism Relat Disord. 2015;21(1):23–30. 25465747 10.1016/j.parkreldis.2014.10.017

[52] Gago MF, Garrett MC, Fonseca MR, et al How do cognitive and axial motor signs correlate in Parkinson's disease? A 6-year prospective study. J Neurol. 2009;256(10):1655–1662. 19471849 10.1007/s00415-009-5174-7

[53] Kozak VV, Chaturvedi M, Gschwandtner U, et al EEG slowing and axial motor impairment are independent predictors of cognitive worsening in a three-year cohort of patients with Parkinson's disease. Front Aging Neurosci. 2020;12:171. 32625079 10.3389/fnagi.2020.00171 PMC7314977

[54] Dubbioso R, Manganelli F, Siebner HR, Di Lazzaro V. Fast intracortical sensory-motor integration: A window into the pathophysiology of Parkinson's disease. Front Hum Neurosci. 2019;13:111. 31024277 10.3389/fnhum.2019.00111 PMC6463734

[55] Baumann A, Nebel A, Granert O, et al Neural correlates of hypokinetic dysarthria and mechanisms of effective voice treatment in Parkinson disease. Neurorehabil Neural Repair. 2018;32(12):1055–1066. 30444176 10.1177/1545968318812726

[56] Kluin K, Gilman S, Foster N, et al Neuropathological correlates of dysarthria in progressive supranuclear palsy. Arch Neurol. 2001;58(2):265–269. 11176965 10.1001/archneur.58.2.265

[57] Di Rauso G, Cavallieri F, Gessani A, et al Speech profile in different clinical PSP phenotypes: An acoustic-perceptual study. Neurol Sci. 2025;46(2):769–774. 39453558 10.1007/s10072-024-07833-w

[58] Suntrup S, Teismann I, Bejer J, et al Evidence for adaptive cortical changes in swallowing in Parkinson's disease. Brain. 2013;136(Pt 3):726–738. 23412935 10.1093/brain/awt004

[59] Takamatsu Y, Matsuda N, Aiba I. The combination of short-step and wide-based gait is a gait characteristic in progressive supranuclear palsy: A retrospective, cross-sectional study. Eur Geriatr Med. 2019;10(5):809–815. 34652693 10.1007/s41999-019-00211-2

[60] Ciolli L, Krismer F, Nicoletti F, Wenning GK. An update on the cerebellar subtype of multiple system atrophy. Cerebellum Ataxias. 2014;1:14. 26331038 10.1186/s40673-014-0014-7 PMC4552412

[61] Rektor I, Bohnen NI, Korczyn AD, et al An updated diagnostic approach to subtype definition of vascular parkinsonism – recommendations from an expert working group. Parkinsonism Relat Disord. 2018;49:9–16. 29310988 10.1016/j.parkreldis.2017.12.030 PMC5857227

[62] Jansen JA, Buurke TJ, van de Venis L, Weerdesteyn V, Keijsers N, Nonnekes J. Narrow-based gait in people with Parkinson's disease: Its mechanisms explored. J Parkinsons Dis. 2025;15(2):329–337. 39973508 10.1177/1877718X241313333 PMC13347439

[63] Frei K . Abnormalities of smooth pursuit in Parkinson's disease: A systematic review. Clin Park Relat Disord. 2021;4:100085. 34316663 10.1016/j.prdoa.2020.100085 PMC8299966

[64] Pinkhardt EH, Jürgens R, Lulé D, et al Eye movement impairments in Parkinson's disease: Possible role of extradopaminergic mechanisms. BMC Neurol. 2012;12:5. 22375860 10.1186/1471-2377-12-5 PMC3306761

[65] Gorges M, Müller HP, Lulé D, Pinkhardt EH, Ludolph AC, Kassubek J. The association between alterations of eye movement control and cerebral intrinsic functional connectivity in Parkinson's disease. Brain Imaging Behav. 2016;10(1):79–91. 25749936 10.1007/s11682-015-9367-7

[66] Li J, Li Y, Chu X, Jiang M, Wu T, Chen X. Reduced maximal range of ocular movements and its response to acute levodopa challenge in Parkinson's disease. Front Aging Neurosci. 2024;16:1368539. 38572152 10.3389/fnagi.2024.1368539 PMC10987739

[67] Corin MS, Elizan TS, Bender MB. Oculomotor function in patients with Parkinson's disease. J Neurol Sci. 1972;15(3):251–265. 5014091 10.1016/0022-510x(72)90068-8

[68] Anderson TJ, MacAskill MR. Eye movements in patients with neurodegenerative disorders. Nat Rev Neurol. Feb. 2013;9(2):74–85. 10.1038/nrneurol.2012.273 23338283

[69] Diotaiuti P, Marotta G, Di Siena F, et al Eye tracking in Parkinson's disease: A review of oculomotor markers and clinical applications. Brain Sci. 2025;15(4):362. 40309816 10.3390/brainsci15040362 PMC12025636

[70] Buch KA, Bouffard MA, Kardon RH, et al Clinical correlation between vertical gaze palsy and midbrain volume in progressive supranuclear palsy. J Neuroophthalmol. 2022;42(2):246–250. 34417776 10.1097/WNO.0000000000001393

[71] Phokaewvarangkul O, Bhidayasiri R. How to spot ocular abnormalities in progressive supranuclear palsy? A practical review. Transl Neurodegener. 2019;8:20. 31333840 10.1186/s40035-019-0160-1 PMC6617936

[72] Yamada Y, Shinkawa K, Kobayashi M, et al Distinct eye movement patterns to complex scenes in Alzheimer's disease and Lewy body disease. Front Neurosci. 2024;18:1333894. 38646608 10.3389/fnins.2024.1333894 PMC11026598

[73] Stuart S, Lawson RA, Yarnall AJ, et al Pro-saccades predict cognitive decline in Parkinson's disease: ICICLE-PD. Mov Disord. 2019;34(11):1690–1698. 31442355 10.1002/mds.27813

[74] Liu P, Basso MA. Substantia nigra stimulation influences monkey superior colliculus neuronal activity bilaterally. J Neurophysiol. 2008;100(2):1098–1112. 18579662 10.1152/jn.01043.2007 PMC2525708

[75] Wenning GK, Stankovic I, Vignatelli L, et al The movement disorder society criteria for the diagnosis of multiple system atrophy. Mov Disord. 2022;37(6):1131–1148. 35445419 10.1002/mds.29005 PMC9321158

[76] Palermo G, Del Prete E, Bonuccelli U, Ceravolo R. Early autonomic and cognitive dysfunction in PD, DLB and MSA: Blurring the boundaries between alpha-synucleinopathies. J Neurol. 2020;267(12):3444–3456. 32594302 10.1007/s00415-020-09985-z PMC7320652

[77] Xu H, Zheng X, Xing X, et al Advances in autonomic dysfunction research in Parkinson's disease. Front Aging Neurosci. 2025;17:1468895. 40144363 10.3389/fnagi.2025.1468895 PMC11937016

[78] Papapetropoulos S, Mash DC. Insular pathology in Parkinson's disease patients with orthostatic hypotension. Parkinsonism Relat Disord. 2007;13(5):308–311. 16962365 10.1016/j.parkreldis.2006.06.009

[79] Wakabayashi K, Takahashi H. Neuropathology of autonomic nervous system in Parkinson's disease. Eur Neurol. 1997;38(Suppl 2):2–7. 10.1159/000113469 9387796

[80] Chung SJ, Bae YJ, Jun S, et al Dysautonomia is associated with structural and functional alterations in Parkinson disease. Neurology. 2019;92(13):e1456–e1467. 30796135 10.1212/WNL.0000000000007181

[81] Udow SJ, Robertson AD, MacIntosh BJ, et al ‘Under pressure': Is there a link between orthostatic hypotension and cognitive impairment in alpha-synucleinopathies? J Neurol Neurosurg Psychiatry. 2016;87(12):1311–1321. 27613160 10.1136/jnnp-2016-314123

[82] McDonald C, Newton JL, Burn DJ. Orthostatic hypotension and cognitive impairment in Parkinson's disease: Causation or association? Mov Disord. 2016;31(7):937–946. 27091624 10.1002/mds.26632

[83] Ruiz Barrio I, Miki Y, Jaunmuktane ZT, Warner T, De Pablo-Fernandez E. Association between orthostatic hypotension and dementia in patients with Parkinson disease and multiple system atrophy. Neurology. 2023;100(10):e998–e1008. 36526431 10.1212/WNL.0000000000201659 PMC9990860

[84] Jones JD, Rahmani E, Garcia E, Jacobs JP. Gastrointestinal symptoms are predictive of trajectories of cognitive functioning in de novo Parkinson's disease. Parkinsonism Relat Disord. 2020;72:7–12. 32058266 10.1016/j.parkreldis.2020.01.009 PMC7179075

[85] Camacho M, Macleod AD, Maple-Grodem J, et al Early constipation predicts faster dementia onset in Parkinson's disease. NPJ Parkinsons Dis. 2021;7(1):45. 34039994 10.1038/s41531-021-00191-w PMC8154963

[86] Counsell C, Giuntoli C, Khan QI, Maple-Grodem J, Macleod AD. The incidence, baseline predictors, and outcomes of dementia in an incident cohort of Parkinson's disease and controls. J Neurol. 2022;269(8):4288–4298. 35307754 10.1007/s00415-022-11058-2 PMC9294013

[87] Gagnon JF, Vendette M, Postuma RB, et al Mild cognitive impairment in rapid eye movement sleep behavior disorder and Parkinson's disease. Ann Neurol. 2009;66(1):39–47. 19670440 10.1002/ana.21680

[88] Boucetta S, Salimi A, Dadar M, Jones BE, Collins DL, Dang-Vu TT. Structural brain alterations associated with rapid eye movement sleep behavior disorder in Parkinson's disease. Sci Rep. 2016;6:26782. 27245317 10.1038/srep26782 PMC4887790

[89] Boeve BF, Silber MH, Saper CB, et al Pathophysiology of REM sleep behaviour disorder and relevance to neurodegenerative disease. Brain. 2007;130(Pt 11):2770–2788. 17412731 10.1093/brain/awm056

[90] Chan PC, Lee HH, Hong CT, Hu CJ, Wu D. REM sleep behavior disorder (RBD) in dementia with Lewy bodies (DLB). Behav Neurol. 2018;2018:9421098. 30018672 10.1155/2018/9421098 PMC6029467

[91] Mao J, Huang X, Yu J, et al Association between REM sleep behavior disorder and cognitive dysfunctions in Parkinson's disease: A systematic review and meta-analysis of observational studies. Front Neurol. 2020;11:577874. 33240202 10.3389/fneur.2020.577874 PMC7677514

[92] Gratwicke J, Kahan J, Zrinzo L, et al The nucleus basalis of Meynert: A new target for deep brain stimulation in dementia? Neurosci Biobehav Rev. 2013;37(10 Pt 2):2676–2688. 24035740 10.1016/j.neubiorev.2013.09.003

[93] Stang CD, Mullan AF, Hajeb M, et al Timeline of rapid eye movement sleep behavior disorder in overt alpha-synucleinopathies. Ann Neurol. 2021;89(2):293–303. 33155696 10.1002/ana.25952 PMC8080353

[94] Vale TC, Barbosa MT, Caramelli P, Cardoso F. Vascular parkinsonism and cognitive impairment: Literature review, Brazilian studies and case vignettes. Dement Neuropsychol. 2012;6(3):137–144. 29213787 10.1590/S1980-57642012DN06030005 PMC5618960

[95] Benítez-Rivero S, Lama MJ, Huertas-Fernández I, et al Clinical features and neuropsychological profile in vascular parkinsonism. J Neurol Sci. 2014;345(1–2):193–197. 25108818 10.1016/j.jns.2014.07.046

[96] Adams RD, Fisher CM, Hakim S, Ojemann RG, Sweet WH. Symptomatic occult hydrocephalus with “normal” cerebrospinal-fluid pressure. a treatable syndrome. N Engl J Med. 1965;273:117–126. 14303656 10.1056/NEJM196507152730301

